# Metabolomics with Nuclear Magnetic Resonance Spectroscopy in a *Drosophila melanogaster* Model of Surviving Sepsis

**DOI:** 10.3390/metabo6040047

**Published:** 2016-12-21

**Authors:** Veli Bakalov, Roland Amathieu, Mohamed N. Triba, Marie-Jeanne Clément, Laura Reyes Uribe, Laurence Le Moyec, Ata Murat Kaynar

**Affiliations:** 1 Clinical Research, Investigation, and Systems Modeling of Acute Illness (CRISMA) Laboratory, Department of Critical Care Medicine, University of Pittsburgh School of Medicine, Pittsburgh, 15261 PA, USA; bakalovv@upmc.edu (V.B.); laurare3@gmail.com (L.R.U.); 2 Laboratoire Chimie, Structures, Propriétés de Biomatériaux et d’Agents Thérapeutiques (CSPBAT), UMR 7244, Université Paris 13, 93000 Bobigny, France; roland.amathieu@aphp.fr (R.A.); mohamed.triba@univ-paris13.fr (M.N.T.); 3 Intensive Care Unit, Jean Verdier University Hospital, Paris 13 University, 93140 Bondy, France; 4 Laboratoire Structure—Activité des biomolécules Normales et Pathologiques (SABNP), U1204, Université d’Evry Val d’Essonne, 91025 Evry, France; mariejeanne.laicheong@univ-evry.fr; 5 Unité de Biologie Intégrative des Adaptations à l’Exercice (UBIAE), EA 7362, Université d’Evry Val d’Essonne, 91025 Evry, France; laurence.lemoyec@univ-evry.fr; 6 Department of Anesthesiology, University of Pittsburgh, Pittsburgh, 15261 PA, USA

**Keywords:** NMR, sepsis, survival, metabolomics

## Abstract

Patients surviving sepsis demonstrate sustained inflammation, which has been associated with long-term complications. One of the main mechanisms behind sustained inflammation is a metabolic switch in parenchymal and immune cells, thus understanding metabolic alterations after sepsis may provide important insights to the pathophysiology of sepsis recovery. In this study, we explored metabolomics in a novel *Drosophila melanogaster* model of surviving sepsis using Nuclear Magnetic Resonance (NMR), to determine metabolite profiles. We used a model of percutaneous infection in *Drosophila melanogaster* to mimic sepsis. We had three experimental groups: sepsis survivors (infected with Staphylococcus aureus and treated with oral linezolid), sham (pricked with an aseptic needle), and unmanipulated (positive control). We performed metabolic measurements seven days after sepsis. We then implemented metabolites detected in NMR spectra into the MetExplore web server in order to identify the metabolic pathway alterations in sepsis surviving *Drosophila*. Our NMR metabolomic approach in a *Drosophila* model of recovery from sepsis clearly distinguished between all three groups and showed two different metabolomic signatures of inflammation. Sham flies had decreased levels of maltose, alanine, and glutamine, while their level of choline was increased. Sepsis survivors had a metabolic signature characterized by decreased glucose, maltose, tyrosine, beta-alanine, acetate, glutamine, and succinate.

## 1. Introduction

Long-term outcomes in sepsis survivors are associated with increased mortality as well as morbidity, such as late cardiovascular events, neuro-cognitive decline, cancer, and metabolic disturbances, yet the mechanisms are not well understood [1]. Sepsis survivors show that sustained inflammation, despite the lack of clinical findings and sustained inflammation, has been associated in clinical studies to unfavorable outcomes [1,2]. A switch of metabolic flux in immune cells from oxidative phosphorylation to aerobic glycolysis (Warburg effect) is a likely mechanism behind the sustained inflammation [3,4].

Inflammatory response is involved in several pathological mechanisms and it is highly integrated into metabolic pathways [5,6]. The single metabolite or pathway-driven approach towards metabolic reprogramming however, carries the risk of not capturing the various parallel processes occurring in organisms surviving sepsis. Recent studies show the importance of using metabolomics in an integrated approach to better understand pathophysiology of sepsis [4,7,8]. Among the analytical techniques used in metabolomics, NMR spectroscopy does not necessitate highly sophisticated sample preparation and allows the use of only a single and unique type of experiment for all metabolites explored. NMR metabolomics have been widely applied in biofluids, cells, tissues or extracts of biological samples, highlighting the metabolic pathways in pathologies such as cirrhosis [9,10], cancer [11], or cardiovascular disease [12]. Metabolic alterations during sepsis control the inflammatory response and can be detected by metabolomics for early diagnosis and outcome prediction in sepsis patients [13].

*Drosophila* has emerged as an ideal model for studying immune response to sepsis. The fruit fly gives us the advantage of exploring long-term outcomes in an intact organism with only an innate immune system over a relatively short time [14,15]. Hence, *Drosophila* provides important insight and is relevant to human innate immune response [16]. In this study, we explored metabolite profiles and metabolic pathways in a novel *Drosophila melanogaster* model of surviving sepsis using NMR [10,11]. We had three experimental groups, unmanipulated, sham and sepsis survivor flies, and metabolites were extracted to perform NMR spectra. This NMR metabolomic study produced metabolomic profiles characterizing each fly group and demonstrating the metabolic adaptation of *Drosophila melanogaster* in response to sepsis.

## 2. Results

**Figure 1:**
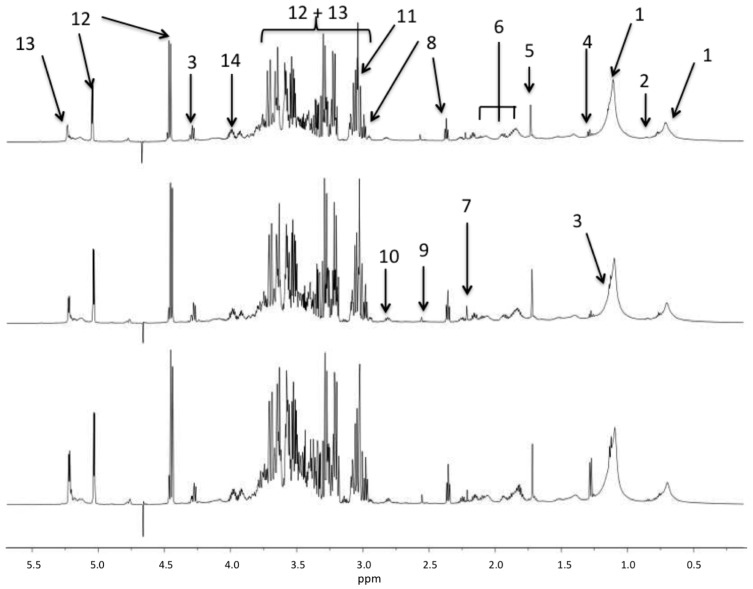
^1^H NMR spectral region between 0.5 and 6 ppm of *Drosophila* samples from the umanipulated (**bottom**), sham (**middle**), and sepsis survivor (**top**) groups. The main resonances are labeled according to metabolite assignments of the Table 1.

Typical spectra obtained from the three groups of flies are shown in the Figure 1. The spectra is dominated by the resonances of carbohydrates from 3.05 to 4.0 ppm and the metabolomic differences are not obvious without the spectra scaling and normalization used for multivariate analysis.

The principal component analysis (PCA) did not show any outlier samples, however it was not able to separate the sample spectra according to the groups, neither to any technical confounding factors such as the phase correction or the day of spectral acquisition.

The partial least squares (PLS) model computed with all samples aimed to separate the three groups, unmanipulated, sham, and sepsis survivors. The model is shown in the supplemental Figure S1. The score plot demonstrates a good separation between unmanipulated, sham, and sepsis survivor groups as confirmed by the R^2^Y and Q^2^ values (0.959 and 0.694, respectively) and the *p*-value of the CV ANOVA test is 0.045. In the PLS model, the first component (horizontal) clearly separates the unmanipulated group from the sepsis survivor group and the second component, the sham group, is separated from the two other groups (Figure S1). The loading plot shows that along the first component, several metabolites are changed in a decreasing order from unmanipulated to sham and to sepsis survivors. The same trend holds true for maltose and glucose, and amino acids such as alanine, beta-alanine, and glutamine.

**Figure 2:**
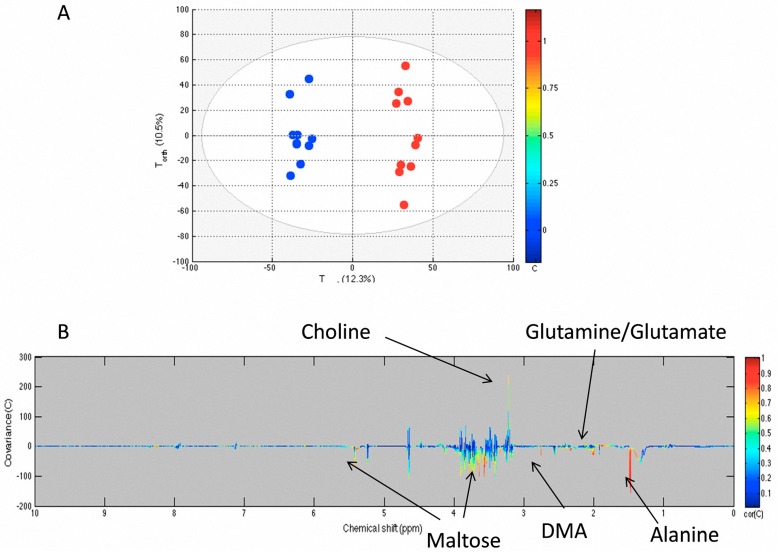
OPLS model comparing unmanipulated (blue) and sham (red) groups. The score plot (**A**) shows the variability of each sample according to their groups with T_pred_ axis representing the predictive axis and T_orth_, the orthogonal axis. Each dot corresponds to a spectrum; The loading plot (**B**) shows the covariance of the spectral bins colored according to the *R* values between the model and the belonging group. Positive signals correspond to metabolites present at increased concentrations in the sham group. Conversely, negative signals correspond to metabolites present at increased concentrations in the unmanipulated group.

We compared NMR-acquired data of sham flies to the unmanipulated flies using an orthogonal projection on latent structures (OPLS) model. Figure 2 shows that the sterile pricking in sham flies directly affects the flies’ metabolome, as the model quality parameters were R^2^Y = 0.982 and Q^2^Y = 0.863. The model was obtained with two components. The Q^2^Y value was higher than the 99th percentile (0.44) of the Q^2^Y values obtained by the validation test with 999 permutations. The loading plot shows that maltose was decreased in the sham group compared to unmanipulated flies but not glucose. The level of choline increased, whereas glutamine and alanine decreased in sham flies compared to unmanipulated flies.

**Figure 3:**
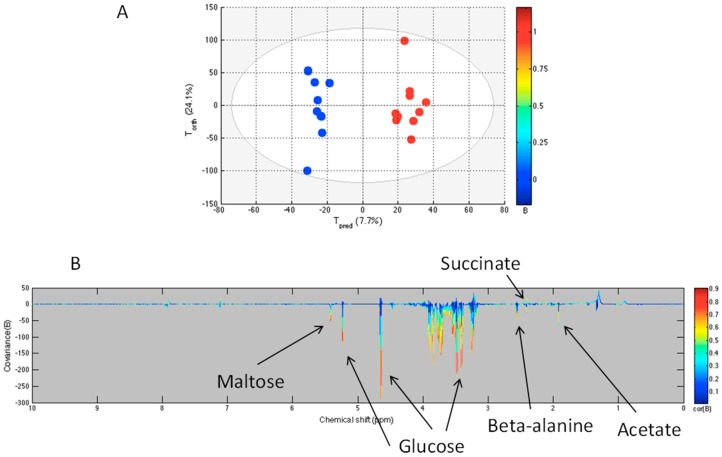
OPLS model comparing sham (blue) and sepsis survivors (red) groups. The score plot (**A**) shows the variability of each sample according to their groups with T_pred_ axis representing the predictive axis and T_orth_, the orthogonal axis. Each dot corresponds to a spectrum. The loading plot (**B**) shows the covariance of the spectral bins colored according to the *R* values between the model and the corresponding group. Positive signals correspond to metabolites present at increased concentrations in sepsis survivors group; negative signals correspond to metabolites present at increased concentrations in the sham group.

Using NMR-acquired data, we compared changes in the metabolome of sham and sepsis survivors using an OPLS computed model. The score plot and the loading plot are presented in Figure 3. The statistical parameters demonstrate a good discrimination between the sham and the sepsis survivors as well as good correlation and predictability with the R^2^Y = 0.968 and Q^2^Y = 0.568. The model was obtained with three components. The Q^2^Y value was higher than the 99th percentile (0.49) of the Q^2^Y values obtained by validation test with 999 permutations. The loading plot shows that several metabolites are decreased in sepsis survivors compared to the sham group, including glucose, maltose, beta-alanine, and acetate (Table 1).

Table 1 summarizes the metabolites identified in the NMR spectra of the three groups and those with higher correlation to the OPLS model, discriminating sham from sepsis survivor groups. Those metabolites were also the metabolites used to construct the Metexplore metabolic pathways. 

**Figure 4:**
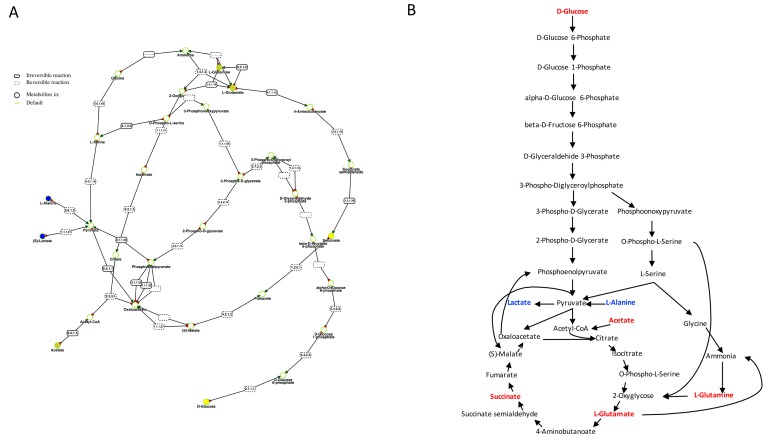
Summary of the metabolic pathways in *Drosophila melanogaster* involved in the metabolic adaptation in sepsis survivors compared to sham. (**A**) is the Metexplore extracted sub-pathway with metabolites down-regulated are colored in yellow and those detected and not modulated are colored in blue; (**B**) shows the metabolites detected not modulated in the metabolomic profile in blue while the metabolites down-modulated are colored in red.

The metabolites detected as discriminant between sham and sepsis survivor flies were used to generate metabolic pathways in Metexplore [17] mapping with two lists of nodes, one only containing the discriminant metabolites (corresponding to |*R*| > 0.5) and a second one with all the metabolites of Table 1. In both cases, the main metabolic pathway containing the highest number of discriminant metabolites includes the reactions of glycolysis and a part of tricarboxylic acid (TCA) cycle (Figure 4). 

## 3. Discussion

In this study, we showed different metabolomics profiles detected by NMR spectroscopy that clearly distinguish between unmanipulated, sham, and sepsis survivor flies. The metabolic pathways differentiating these three groups are mainly those of glycolysis and carbohydrate consumption in relation to tricarboxylic acid (TCA) cycle and amino acid utilization. The involvement of these metabolic pathways was visualized and confirmed their enzymatic and genetic possibility by the Metexplore metabolic networks using the *Drosophila melanogaster* genome-based method. 

The cellular and molecular mechanisms and signaling pathways involved in *Drosophila* wound repair and infection are widely described [18]. While the biochemistry and metabolic descriptions were performed earlier, the NMR metabolomic investigation provides new insight into metabolic pathways modulation in response to stress. With the benefit of this multiparametric and non-targeted method, several pathways were explored in a single experiment.

As a first step, the metabolomic OPLS model discriminating between unmanipulated and sham flies demonstrated that the needle pricking may be responsible for sterile inflammation and trigger changes in the metabolic pathways including a decrease of maltose content, but not glucose, and also a decrease in amino acids, including glutamine and alanine, and an increase of choline. Consequently, to investigate metabolic changes following sepsis, we did undertake a comparison of metabolomic profiles of sham and sepsis surviving *Drosophila*. The OPLS model demonstrates that sepsis survivor flies are undergoing a metabolomic response distinctive from the sterile inflammation observed in sham flies. Maltose is significantly decreased in sepsis survivors when compared to the sham group. Moreover, low glucose level suggests the involvement of carbohydrates in the metabolic changes during the inflammatory response in sepsis survivors. The amino acids involved in the discrimination are glutamine/glutamate, beta-alanine, and tyrosine, while alanine was not a discriminant metabolite. 

In *Drosophila*, maltose is the product of starch hydrolysis. This disaccharide can be hydrolyzed into glucose, thus it may be considered as a glucose stock. Activated immune cells demonstrate the Warburg effect characterized as a metabolic switch towards aerobic glycolysis and increased glucose consumption [19]. In our previous work [14], we showed sustained inflammation and decreased glucose storage in sepsis survivors supporting carbohydrate metabolomics profiles. Increased bioenergetic demand during the inflammatory response can explain decreased maltose storage in sham as well as in sepsis survivors. Higher inflammatory response in sepsis survivors, in addition to decreased maltose storage, could contribute to increased glucose consumption.

In sepsis survivors and sham groups, the reduction of glutamine/glutamate could be explained by the high glucose consumption as these amino acids are direct energy suppliers through de-amination to produce alpha-keto-glutarate (αKG), a TCA cycle metabolite [20]. This process is often observed in the setting of high glucose demand. 

In addition, the sham group had decreased alanine levels compared to unmanipulated flies, however, alanine is not a discriminate metabolite in sepsis survivors when compared to sham group, suggesting that the level of alanine utilization remains high after septic injury and thus, participates in the energy supply.

Interestingly, lactate level did not participate in the group discrimination, whereas it was previously reported to be high in sepsis surviving flies using colorimetric assay method [14]. Lactate does not discriminate sham from unmanipulated, nor sepsis survivors from sham flies, while lactate and LDH activity were increased in sepsis survivors in our previous study [14]. It is noticeable that NMR spectroscopy used here is only sensitive to free lactate and not to lactate bound to proteins or macromolecules, the latter is detected by biochemistry methods [21].

The amino acid profile was also different in all three groups. Tyrosine, a precursor of DOPA, Dopamine, and other amines, was lower in sepsis survivors. Melanization plays an important role in *Drosophila* immune defense, and during this process L-DOPA synthesized from tyrosine is catalyzed by phenoloxidase in order to produce melanin [22]. Decrease of tyrosine in sepsis survivors and the sham group could be explained by consumption during the melanization process.

Sterile injury provoked by pricking in sham flies contributes to the phospholipids destruction of the cell membrane, subsequent synthesis phosphatidylcholine in order to repair the cell membrane can explain changes in choline level [23,24]. The resonance at 2.75 ppm was attributed to dimethylamine as corresponding to the chemical shift and coherent with no correlation found in the 2DTOCSY (2-dimensional total correlation spectroscopy) spectra acquired. Dimethylamine is decreased in the profile of sham flies when compared to the unmanipulated group and it remains low and was not a discriminant metabolite in sepsis survivors. Dimethylamine, an osmotic amine found in the kidney of human and fish, could protect from dehydration susceptibility after the wound formation.

In *Drosophila*, beta-alanine is part of carcinine dipeptide (beta-alanine-histamine), is mainly localized in the head and thorax [25] and plays an important role in cuticule melanization [26] and signaling in photoreceptor synapse [27]. Similarly to tyrosine, decrease of beta-alanine could be explained by its consumption during the melanization process.

Acetate and succinate are discriminant metabolites and decreased in sepsis survivors compared to the sham group. Acetate is directly related to energy metabolism as two sources for this NMR resonance may be evoked. First, acetate resonance may be produced by the free ketone body derived from lipid beta-oxidation. Lipid consumption takes place when carbohydrate stocks are low. In the sham flies, the acetate content is higher than in sepsis survivors, the latter containing less carbohydrate (maltose and glucose). This profile did let us hypothesize that lipid oxidation could be higher in sham flies than in sepsis survivors in which the carbohydrate pathway is favored. The second origin of acetate peaks in NMR spectra is the acetyl moiety of acetyl-CoA, which is also directly involved in energy metabolism as the first entry in TCA after glycolysis. Consequently, decrease of acetate in sepsis survivors may be related to a decrease of carbohydrate content. 

Sepsis surviving flies had decreased succinate concentration compared to the sham group. Succinate is a TCA cycle metabolite and plays an important role in the production of ATP; in addition, succinate contributes to the stabilization of transcription factor hypoxia-inducible factor-1α (HIF-1α) with subsequent transcription of proinflammatory cytokines [28,29]. Recent works also suggest a signaling role for succinate (both paracrine as well as autocrine fashion) in inflammation [30,31]. In cancer cells, as well as in LPS stimulated macrophages demonstrating Warburg effect, increased succinate oxidation promotes a proinflammatory state, and thus contributes to tumor growth or inflammation. Once oxidized, succinate is converted to fumarate, thus decreasing its concentrations. 

Our work has some limitations. First of all, due to the small body size and difficulty in isolation of a sufficient amount of hemolymph or specific tissue in *Drosophila*, measurement of metabolites in the whole body is the most practical approach. While this is a limitation of our study, metabolomic profiles of the whole body provide important information about metabolic processes in the body. In addition, we used an orally available antibiotic (linezolid) to achieve survival from sepsis. Although, linezolid protects flies from S. aureus infection without evident toxicity [32], in humans is known to impair mitochondrial protein synthesis [33,34] and produce hyperlactatemia with chronic use [35,36]. In order to eliminate the possible contribution of linezolid to the metabolic profile, all three groups were treated identically for 24 h only. In addition, in order to collect flies for metabolomic analysis we used CO_2_ anesthesia. Although seven minutes of CO_2_ anesthesia can cause metabolic changes [37], we used less than one minute of CO_2_ anesthesia and all groups were exposed to same conditions.

## 4. Materials and Methods

### 4.1. Experimental Design

We used a model of percutaneous infection in *Drosophila melanogaster* to mimic sepsis and treated with orally available linezolid [14]. We followed immune and metabolic outcomes over a seven-day course. We had three experimental groups: (a) unmanipulated; (b) sham; and (c) infected followed by antibiotic treatment (sepsis survivors).

### 4.2. Drosophila Melanogaster Strains and Maintenance

We raised the flies at 23 °C, 60% humidity, and 12-h light/dark cycle on standard cornmeal-yeast medium, changed every three to five days. We used two or three day old male flies. Wild-type (WT) Canton S were obtained from Bloomington stock.

### 4.3. Fly Infection and Treatment

We separated flies in 10 vials of 50 flies for each group. One group was infected with *Staphylococcus aureus* and treated with oral linezolid (sepsis survivors), the second group was pricked with an aseptic needle (sham), and the last group was left as a control (unmanipulated) [14]. To eliminate possible side effects of linezolid, we treated sham and unmanipulated files with antibiotic for 24 h similarly to the sepsis survivors group. 

### 4.4. Nuclear Magnetic Resonance Spectroscopy

We performed metabolomic measurements one week after infection. Each sample of 50 flies was homogenized on ice in 1.5 mL tubes using plastic pestles in 400 μL 50% acetonitrile, then centrifuged at 17,000 rpm, at +4 °C for 10 min and 350 μL of the supernatant was snap frozen in liquid nitrogen and then lyophilized (Thermo-Savant SPD1010) and stored at −80 °C until NMR experiments. Ready samples were transferred on dry ice to the NMR laboratory.

For NMR spectroscopy, the samples were solubilized in 60 μL deuterium oxide (D_2_O) and introduced into 1.7 mm capillary tubes further placed in capillary holder containing D_2_O. The proton spectra were acquired at 600 MHz and 295 K with a Bruker Avance spectrometer (Wissembourg, France) using a cryoprobe. The acquisition sequence was a NOESY1D (Nuclear Over-Hauser Spectroscopy One Dimension) sequence including the water suppression, a 60 ms spin-lock, 90° pulse, and 3 s relaxation delay. The number of transient was 64 on 32 K data points. The spectra were obtained during two consecutive days and samples were randomly ordered. The free induction decay (FID) was processed in the NMR pipe using an in-house script for automation of Fourier transform with a 0.3 Hz line broadening, baseline correction, and calibration using acetate signal at 1.92 ppm while spectra were phased manually. The 30 spectra were binned into 0.001 ppm bins with elimination of the water region between 4.6 and 5 ppm to produce the X matrix, which is used for multivariate statistical analysis. 

The NMR spectra attribution were performed according to our prior works [9], the Human Metabolome Database (HMDB) [38], and previous reports on NMR metabolomics with *Drosophila* [39,40].

### 4.5. Statistical Analysis

For multivariate analysis, NMR data collected in the X matrix are normalized along the spectral variables using the probabilistic quotient method and the variables are centered normalized. The principal component analysis (PCA), projection on latent structure (PLS), and orthogonal projection on latent structure (OPLS) analyses were performed using an in-house MATLAB (2011b) code (Mathworks, Natick, MA, USA) based on the Trygg and Wold method [41] as previously described [11]. The PCA was used in order to detect any confounding factors (spectroscopy acquisition day, phase) and any outlier samples. The PLS method using the three groups as supervising factors was applied in order to detect the possible discrimination of the three conditions. Finally, OPLS models were used to compare the groups two by two. The quality of the model is assessed by the correlation coefficient (R2Y), the predictive coefficient (Q2Y), R2Y = 1 indicates perfect description of the data by the model, whereas Q2Y = 1 indicates perfect predictability. The Q2Y was computed with the “leave-one-out” cross-validation method [42]. As another mean for internal validation of the O-PLS models, a permutation test (999 permutations) was performed [43]. This evaluated whether the O-PLS models, built with the samples, were significantly better than any other O-PLS model obtained by randomly permuting the original samples attributes.

The results are presented as a score plot showing the discrimination between groups and as a loading plot colored according to the correlation of each bucket to the model. Each point in the score-plot represents the projection of the NMR spectrum (and thus a fly sample) on the two first components of OPLS model, on the predictive (Tpred, horizontal axis), and the first orthogonal component of the model (Torth, vertical axis). The loading plot represents the covariance between the Y-response matrix and the signal intensity of the various spectral domains. Colors were also used in the loading plot depending on the correlation between the corresponding bucket intensity and Y variable. The metabolites were considered as discriminating metabolites when corresponding to the buckets with a correlation over or equal to 0.5.

### 4.6. Metabolic Pathways Analysis

The metabolites detected in NMR spectra and discriminant metabolites were implemented into the MetExplore web server [44] in order to identify the metabolic pathways modified in the sepsis surviving *Drosophilae*. The metabolic network of *Drosophila melanogaster* is based on 85 metabolic pathways including 185 reactions. The representation of the metabolic pathways involved in the metabolic process detected in sham and sepsis survivors was produced by the MetExplore system and included in classical pathway representation after identification.

## 5. Conclusions

Our NMR metabolomic approach in a *Drosophila* model of recovery from sepsis showed two different metabolomic signatures of inflammation. The first signature is in the sham group, which seems to be related to non-septic inflammation and the second one in the sepsis survivors associated with a septic inflammation triggered by septic injury. Sepsis survivors had a metabolic signature characterized with decreased glucose, tyrosine, beta-alanine, and succinate. Further studies investigating differences between non-surviving and surviving flies at different time-lapse and tissue specific metabolomic profiling would be of a high importance for better understanding the pathophysiology of sepsis recovery and designing future therapeutics for patients surviving sepsis.

## Figures and Tables

**Table 1:** Main metabolites detected in 1H-NMR spectra with their chemical shift and labels presented in Figure 1. The correlation coefficient R between the OPLS model and the signal intensity is given for the model computed between sham and sepsis survivors.

Peak Labels in Figure 1	Chemical Shift	Assignment	|*R*| > 0.5 Sham vs. Sepsis Survivors
1	0.9; 1.28	Fatty acid methyl and methylene moieties	
2	0.98–1.05	Leucine, Isoleucine, Valine	
3	1.33	Lactate	
4	1.48	Alanine	
5	1.92	Acetate	−0.525
6	2.03; 2.16; 2.34	Glutamine + Glutamate	−0.542
7	2.41	Succinate	−0.708
8	2.56	Beta-alanine	−0.676
9	2.75	Dimethylamine (DMA)	
10	3.01	Lysine	
11	3.22	Choline	
12	3.23–4.0; 4.62; 5.23	Glucose	−0.725
13	3.5–4.0; 4.45; 5.42	Maltose	−0.701
14	4.1; 4.15	Glycerol	
not shown	7.12	Methyl-histidine	
not shown	7.94	Histidine	
not shown	6.89; 7.20	Tyrosine	−0.667

## Supplementary Materials

The following are available online at www.mdpi.com/2218-1989/6/4/47/s1, Figure S1. PLS model computed with the three groups of *Drosophila melanogaster*; Figure S2. MetExplore extracted sub-pathways relating the highest number of metabolites from the Table 1.
