# Supplementary material for: Metabolomics with Nuclear Magnetic Resonance Spectroscopy in a Drosophila melanogaster Model of Surviving Sepsis
Source: Metabolites. 2016 Dec 21;6(4):47. doi: 10.3390/metabo6040047 (PMC5192453; doi:10.3390/metabo6040047)
Supplement: Supplementary file 1 [file metabolites-06-00047-s001.pdf]

Supplementary Materials: Metabolomics with Nuclear Magnetic Resonance Spectroscopy in a *Drosophila melanogaster* Model of Surviving Sepsis

Veli Bakalov, Roland Amathieu, Mohamed N. Triba, Marie-Jeanne Clément, Laura Reyes Uribe, Laurence Le Moyec and Ata Murat Kaynar

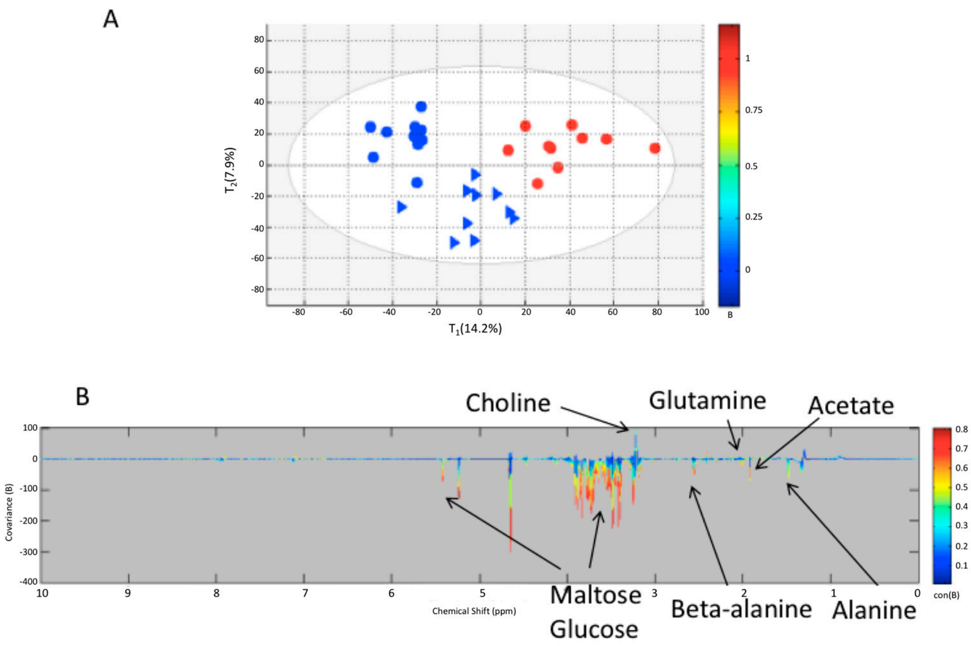

Figure S1. PLS model computed with the three groups of *Drosophila melanogaster*. The score plot (**A**) shows the variability of each sample according to their groups; The loading plot (**B**) shows the covariance of the spectral bins colored according to the *R* values between the model and the group belonging. Blue dots: unmanipulated; blue triangles: sham; Red dots: sepsis survivors.

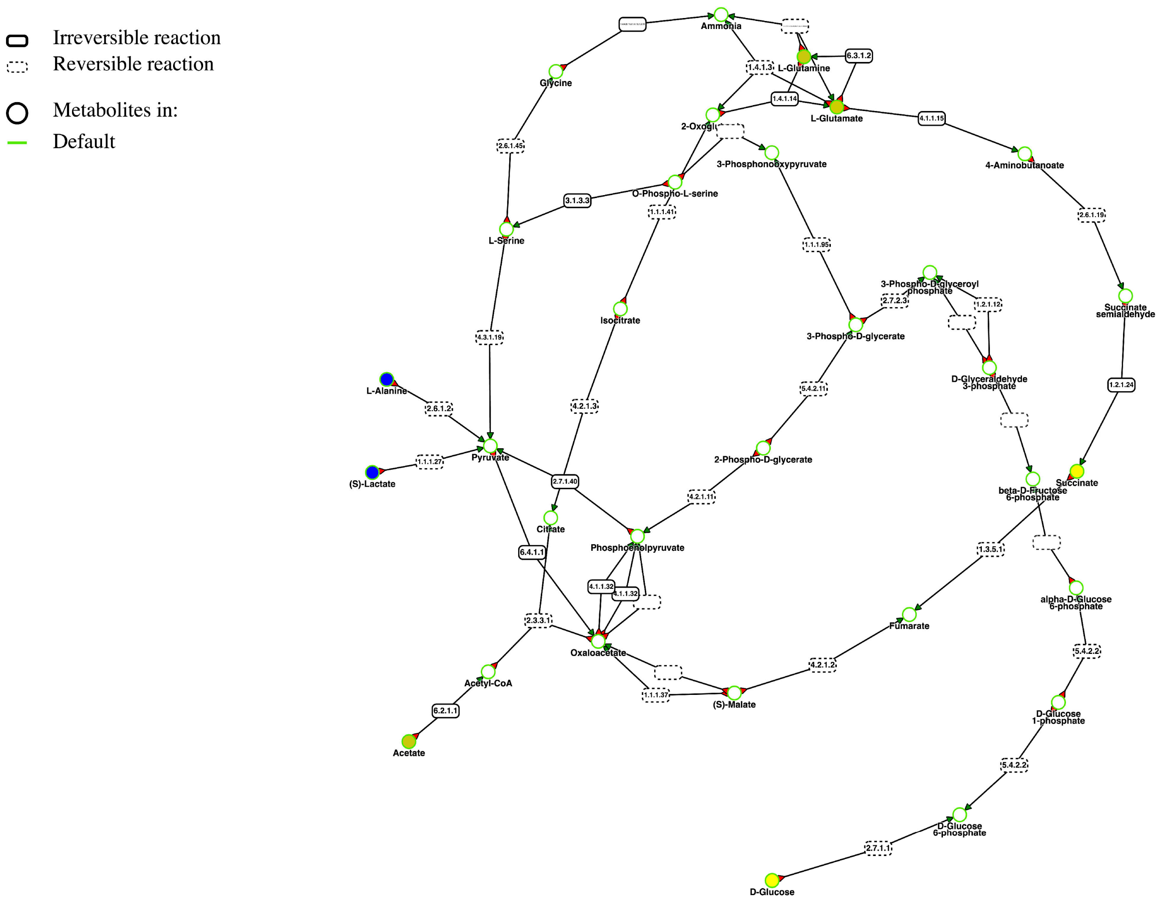

Figure S2. MetExplore extracted sub-pathways relating the highest number of metabolites from the Table 1. The metabolites, in OPLS model comparing sham to sepsis survivors, down-regulated are colored in yellow and those detected not modulated colored in blue.
